# Factors that affect the traumatic childbirth perceptions of midwifery and nursing students: The case of Turkey

**DOI:** 10.18332/ejm/138596

**Published:** 2021-08-05

**Authors:** Filiz Aslantekin-Özçoban, Hülya Türkmen, Hacer Yalnız-Dilcen

**Affiliations:** 1 Department of Midwifery, Faculty of Health Sciences, Balikesir University, Balikesir, Turkey; 2 Anatolian Midwives Association, Ankara, Turkey

**Keywords:** traumatic childbirth perception, anxiety level, self-efficacy, prospective nurse-midwife

## Abstract

**INTRODUCTION:**

Birth is a natural and joyful situation as well as a process that contains surprise situations that do not go well. Caregivers at birth are affected by this process. Especially when faced with difficult births, it can have an intense psychological effect and a perception of traumatic birth can occur. Although there is research about midwives on this subject, there are very few studies about students who are becoming midwives. The aim of this study was to determine the factors that affect the traumatic childbirth perceptions of midwifery and nursing students.

**METHODS:**

The study was carried out with 480 students of midwifery and nursing. The data were collected by using a Personal Information Form, the Rosenberg Self-Esteem Scale, Self-Efficacy Scale, Traumatic Childbirth Perception Scale, and State-Trait Anxiety Inventory.

**RESULTS:**

The traumatic childbirth perception levels were very low in 7.3% of the participants, low in 26.9%, moderate in 37.9%, high in 21.5% and very high in 6.9%. The regression analysis revealed a significant relationship between traumatic childbirth perceptions and the parameters of satisfaction with the department studied, fear of childbirth, defining childbirth as a difficult and painful process, and history of complicated birth in the family. There was also a significant relationship between traumatic childbirth perceptions and the parameters of trait anxiety and general self-esteem.

**CONCLUSIONS:**

Traumatic childbirth perceptions increased as the state and trait anxiety levels and self-esteem levels increased, while they decreased as the self-efficacy levels increased.

## INTRODUCTION

Midwifery is an emotionally difficult profession as it requires professional responsibility for the physical and psychological safety of the mother and the infant^1,2^. While midwives usually witness positive and morale-increasing births, they also encounter traumatic and saddening events. In this context, it is seen that the number of studies that reveal that midwives are under risk of psychological problems is becoming increasingly higher^3-9^.

Midwifery students who provide care during their education in delivery rooms, just like midwives, also feel empathic emotions when they encounter a traumatic event during delivery, and they become defenceless against secondary traumatic stress as a result of this^10^. This situation leads students under a heavy psychological load and to experience heightened perceptions of traumatic childbirth^11^. In addition to this, there are a lot of factors that affect the perception of childbirth as traumatic, while the leading ones among these include the personality characteristics of the individual, their childbirth experience, the childbirth stories that they have heard, the views of society on childbirth, cultural values, and exposure to negative examples of childbirth included in the media^12,13^. Moreover, it is believed that self-esteem, self-efficacy and anxiety issues would also affect the traumatic childbirth perceptions of nursing and midwifery students. The youth period of prospective nurses and midwives is a turning point in terms of development of their self-esteem^14^. Midwives and nurses who have a positive self-concept may change the health of women towards a positive direction^15^. Individuals with low self-efficacy believe that their work is even more difficult than it actually is. This type of thinking increases anxiety and stress, narrowing the perspective required to solve a problem in the best way^16^. Among the reasons why midwifery students do not want to give vaginal birth is that fear of delivery results in an inability to cope with labor pain. In this context, self-efficacy is an important factor considering its relationship with belief in overcoming events^17^. The probability that a set of risks that may arise during labor will harm the infant is a significant source of stress and anxiety for women^18^. High anxiety levels of prospective midwives may lead them to perceive childbirth as traumatic. Anxiety and traumatic stress may also affect the empathic care provided by a practitioner. This is especially important in the context of midwives and midwifery students, because the care provided by midwives and midwifery students may affect the perception of the mother about her childbirth experience^19^.

Studies in the US^3^ and the UK^6^ determined that midwives may develop symptoms of post-traumatic stress disorders (PTSD) after exposure to childbirth trauma. PTSD symptoms in midwives are an important issue due to their probable negative outcomes in terms of care^11^. There is evidence that healthcare professionals who report PTSD symptoms provide care with an emotionally detached approach^20-22^. In this context, for midwives and midwifery students, exposure to trauma and its effects may influence their relationships with the women under their care and decrease their decision-making skills^11^. Traumatic perception of childbirth even in students of nursing and midwifery may affect the reproductive and childbirth preferences of the group of women for whom they provide care. However, at this point, very little is known about the experiences of students regarding traumatic events and their effect on the psychological health of this labor group^6^. To guide evidence-based practices, it is needed to carry out research for assessing the traumatic childbirth perceptions of midwifery and nursing students and determine the influencing factors. To the best of our knowledge, there has been no study on the effects of the self-esteem, self-efficacy and anxiety status of prospective midwives and nurses, who witness childbirth, on their traumatic childbirth perceptions. In this context, this study aimed to determine the factors that affect the traumatic childbirth perceptions of midwifery and nursing students and influencing factors.

Our study’s research question was: ‘What are the factors affecting the traumatic birth perceptions of midwifery and nursing students?’.

## METHODS

### Design and participants

This is a descriptive and cross-sectional study. It was carried out between 1 April and 20 May 2018 in the Department of Midwifery and Nursing, Health Sciences School of Balikesir University, Turkey. There were 1076 students enrolled in the department. Using the Epi Info Statcalc software, sampling of unknown universe, the sample size with the error rate of 0.05 and a 95% confidence interval was calculated as 283. The criteria for sample inclusion were: being a midwife and nursing student and volunteering to participate in the study. The sample included 480 students who agreed to participate in the study. The sample size was increased to improve the quality of the results. Before the questionnaire was distributed, the purpose of the research was explained to the students and that their personal information would remain confidential, before they signed voluntary consent forms. Data collection forms were filled in the classroom and took about 10–15 minutes.

### Instrument

The data were collected using a Personal Information Form, the Rosenberg Self-Esteem Scale (RSES), Traumatic Childbirth Perception Scale, General Self-Efficacy Scale and State-Trait Anxiety Inventory.

#### Personal information form

This form consisted of a total of 41 questions on the sociodemographic, obstetric and gynecological characteristics of the participants^3-8,10-12^.

#### The Rosenberg Self-Esteem Scale (RSES)

In the scale developed by Morris Rosenberg and tested for validity and reliability in Turkish by Çuhadaroğlu: a score 0–1 corresponds to high self-esteem, 2–4 moderate selfesteem, and 5–6 points low self-esteem. RSES consists of twelve dimensions, and the first ten items measure selfesteem. In the scoring process, low scores are associated with high self-esteem, while high scores are associated with low self-esteem. Cronbach’s alpha coefficient of the scale was 0.85^23^ and it was determined as 0.59 in this study. This study also used the first 10 items of the scale to determine the participants’ self-esteem levels^23^.

#### The Traumatic Childbirth Perception Scale (TCPS)

The scale that was developed by Yalnız et al.^24^ includes 13 items, and as the score in the scale increases, the level of perceiving childbirth as traumatic also increases. The minimum possible score on the scale is 0, while the maximum score is 130. A total score of 0–26 points corresponds to very low, 27–52 low, 53–78 moderate, 79–104 high, and 105–130 very high level of traumatic perception of childbirth. Cronbach’s alpha coefficient of the scale was 0.89^24^ and it was determined as 0.90 in this study.

#### The General Self-Efficacy Scale (GSES)

The scale that was developed by Sherer et al.^25^ was tested for validity and reliability in Turkish by Yıldırım and İlhan. The total score in the scale varies in the range 17–85, while higher scores indicate increased beliefs of self-efficacy. The Cronbach’s alpha coefficient of the scale was 0.80^25^ and it was determined as 0.82 in this study.

#### The State-Trait Anxiety Inventory (STAI)

The scale that was developed by Spielberger et al.^26^ was tested for validity and reliability in Turkish by Öner and Le Compte. It is a Likert-type scale that measures levels of state and trait anxiety separately with twenty questions for each. The State Anxiety Inventory (SAI) is a highly sensitive instrument in assessment of emotive reactions that change suddenly. The Trait Anxiety Inventory (TAI), on the other hand, measures the permanence of the anxiety the person is likely to experience in general. In the scale, direct statements express negative emotions, while inverse statements express positive emotions. Kuder Richardson reliability rates were 0.94–0.96 for the State Anxiety Subscale (STAI-S); item correlations reliability rates were 0.42–0.85; and test-retest reliability rates 0.26–0.68. The Cronbach’s alpha internal consistency coefficient in this study was 0.84 for TAI and 0.91 for SAI.

### Statistical analysis

The dependent variable of the study is the perception of traumatic birth. Its independent variables were determined as general self-efficacy, self-esteem, and anxiety. Kolmogorov Smirnov test was used to examine whether or not the data were normally distributed. Accordingly, the data on trait anxiety were normally distributed, while the data on state anxiety, self-efficacy, self-esteem and traumatic childbirth perception scores were non-normally distributed. The relationships between the characteristics of the participants and their levels of traumatic childbirth perceptions were examined by using Mann-Whitney U test and Kruskal-Wallis test. The relationship between the scores of the Traumatic Childbirth Perception Scale and the scores of the General Self-Efficacy Scale, Rosenberg Self-Esteem Scale and State-Trait Anxiety Inventory were analyzed by using Pearson’s r and Spearman’s r_s_ correlation tests. The variables that affected traumatic childbirth perceptions were determined by multiple linear regression analysis. The type I error level in the study was accepted as p<0.05.

## RESULTS

The mean age of the participants was 19.96±1.75 years (range: 13–29). The mean state anxiety score was 41.15±10.47 (range: 20–74), the mean trait anxiety score was 45.28±9 (range: 23–73), the mean self-efficacy score was 61.33±9.72 (range: 33–85), the mean self-esteem score was 19.69±4.03 (range: 10–32), and the mean traumatic perception score was 64.26±27.06 (range: 5–220). The traumatic childbirth perception levels were very low in 7.3% of the participants, low in 26.9%, moderate in 37.9%, high in 21.5%, and very high in 6.9%.

Table 1 shows the relationships between the traumatic childbirth perceptions of the participants and their descriptive characteristics and childbirth-related experiences. The traumatic childbirth perception levels of the students who were not satisfied with the department they studied at were significantly higher (p=0.017). The traumatic childbirth perception levels of those who had a fear of childbirth and defined childbirth as a difficult and painful process were significantly higher (p=0.000). Those with a preference of childbirth via normal vaginal delivery had significantly lower traumatic childbirth perception levels, while those who would prefer cesarean section births had higher levels (p=0.000). The traumatic childbirth perception levels of those who experienced stress and fear in their first delivery room experience were significantly higher (p=0.001). The participants who had a history of complicated birth in their families had significantly higher levels of traumatic childbirth perceptions (p=0.007).

**Table 1:** Relationships between the traumatic childbirth perceptions of the participants and their descriptive characteristics and childbirth-related experiences

*Characteristics*			*Traumatic childbirth perception*
	*n*	*%*	*Mean±SD (Median)*
Type of high school graduates*			
Vocational high school of health	119	24.8	60.17±25.72 (60)
Other	361	75.2	65.61±27.39 (65)
p			0.065
Satisfaction with the department*			
Satisfied	393	82.4	62.47±25.43 (62)
Dissatisfied	84	17.6	71.5±32.52 (72.5)
p			**0.017**
**Have experienced a traumatic event**			
Yes	102	21.4	68.31±25.32 (68)
No	376	78.6	63.15±27.51 (62)
p			0.054
Have taken delivery course*			
Yes	266	55.4	64.54±27.91 (64)
No	214	44.6	63.91±26.03 (63)
p			0.822
Is afraid of childbirth*			
Yes	365	76	69.61±24.97 (69)
No	115	24	47.26±26.5 (39)
p			**0.000**
What is the process of childbirth like?*			
Difficult and painful	370	77.1	66.81±25.99 (66)
Normal and happy	110	22.9	55.68±28.89 (48)
p			**0.000**
Delivery preference**			
Normal vaginal delivery	280	58.3	58.81±26.47 (59)
Cesarean section	47	9.8	76.44±23.36 (75)
Epidural anesthesia	24	5	75.16±30.11 (78)
Water birth	129	26.9	69.62±26.14 (71)
p			**0.000**
Emotion felt in first delivery room experience**			
Comfortable	11	4.1	46.54±29.41 (37)
Excited	121	45.5	64.49±24.46 (62)
Stressed	19	7.1	68.63±28.18 (73)
Afraid	39	14.7	76.97±37.22 (76.5)
Confused	23	8.6	62.43±19.41 (58.5)
A different feeling	53	19.9	58.69±27.37 (62)
p			**0.001**
There is a close person who experienced complications in normal delivery*			
Yes	164	34.2	65.5±28.62 (65.5)
No	316	65.8	63.62±26.24 (63)
p			0.327
There is a close person who experienced complications in cesarean section delivery*			
Yes	179	37.3	66.41±29.48 (65.5)
No	301	62.7	62.99±25.48 (63)
p			0.310
History of difficult birth in the family*			
Yes	172	35.8	68.15±24.67 (67.5)
No	308	64.2	62.09±28.11 (61)
p			**0.007**

* Mann-Whitney U test.

** Kruskal-Wallis test. SD: standard deviation.

As a result of the analysis that was carried out, it was observed that traumatic childbirth perceptions increased as the state and trait anxiety levels and self-esteem levels increased, while they decreased as the self-efficacy levels increased (Table 2).

**Table 2:** Correlation coefficients between the scores of the traumatic childbirth perception scale and the scores of the state-trait anxiety inventory, general self-efficacy scale and Rosenberg self-esteem scale

*Variable*	*Pearson r/Spearman’s r_s_*
SAI	0.293**
TAI	0.316**
GSES	-0.316**
RSES	0.230**

** p<0.01.

In the study, a multiple linear regression analysis was carried out on the independent variables that affected traumatic childbirth perceptions (Table 3). As a result of the analysis, a significant relationship was found between traumatic childbirth perceptions and the variables of satisfaction with the department, fear of childbirth, defining childbirth as a difficult and painful process, and history of difficult birth in the family that explained 0.5% of the total variance (R^2^=0.058). Additionally, there was a significant relationship between traumatic childbirth perceptions and the variables of trait anxiety and general self-efficacy that explained 14% of the total variance (R^2^=0.140).

**Table 3:** Multiple linear regression analysis of the independent variables that affected traumatic childbirth perceptions

*Variable*	*B*	*t*	*p*	*95 % CI*	
				*Lower*	*Upper*
**Model 1**					
Satisfaction with the department	7.220	2.232	**0.000**	0.861	13.58
Fear of childbirth	-1.961	-2.386	**0.017**	-3.576	-0.346
What kind of a process childbirth is	-6.918	-2.619	**0.009**	-12.110	-1.726
Feeling in first delivery room experience	-0.008	-0.018	0.986	-0.845	0.830
History of difficult birth in the family	-5.227	-1.977	**0.049**	-10.422	-0.031
R=0.241 R^2^=0.058 Durbin-Watson = 1.97 6 (p<0.0001)					
**Model 2**					
SAI	0.243	1.738	0.083	-0.032	0.517
TAI	0.370	2.055	**0.040**	0.016	0.724
GSES	-0.538	-3.601	**0.000**	-0.832	-0.244
RSES	0.352	1.059	0.290	-0.301	1.006
R=0.375 R^2^=0.140 Durbin-Watson = 1.849 (p<0.000)					

B: regression coefficient. t: degrees of freedom. p: significance level.

## DISCUSSION

The behaviors of individuals who provide healthcare services may have positive or negative outcomes. To prevent development of potentially greater problems, we need to understand the ramifications of traumatic childbirth^27^. Most studies on this topic are focused on pregnant women or women giving birth. However, there are very few studies on fears of childbirth and birth preferences among young women who may become mothers in the future and use maternal healthcare services. To the best of our knowledge, there has been no study that investigated the traumatic childbirth perceptions of university students with a scale unique to a society as in the case in this study.

In this study, it was found that the satisfaction of the participants with their department decreased their traumatic childbirth perceptions significantly. In the study by Ulusoy et al.^28^, approximately a quarter of the participants were found to already dislike the profession, and it was stated that this may be a reason for students’ dissatisfaction. Thus, individuals who embrace being midwives or nurses, and like the profession, will have interest and knowledge in and tendency towards the process of childbirth.

One of the most important factors that lead to the act of birth to be perceived as traumatic is fear of childbirth^29^ In the study, the traumatic childbirth perceptions of the participants who had fears of childbirth and defined this process as difficult and painful were found to be higher. In Stoll et al.^30^ study, university students with high levels of fear of childbirth defined the process as painful and scary. In the study by Palumbo et al.^31^ which investigated the status of pre-university young people regarding childbirth, it was reported that young people found childbirth to be a normal event in life and described it as painful, scary, stressful and miraculous. In another study which examined the childbirth-related fears of university students, it was found that one seventh of female students who had not experienced childbirth had a fear of it, while this fear was severe in one fourth^32^. In this context, the findings showed that the process of birth constitutes a concern of having a negative experience for the mother and the infant among both women who have experienced pregnancy and childbirth and those who have not. It is believed that this situation may create a tendency towards perceiving childbirth to be traumatic among women.

Among the participants, those who preferred normal vaginal delivery had low levels of traumatic childbirth and state anxiety, while those who preferred a water birth or cesarean section birth, had high levels. In a study which investigated the birth preferences of male and female students, it was found that cesarean section births were preferred due to fear of childbirth and low self-esteem about vaginal delivery^33^. A study reported that students who had high anxiety levels preferred cesarean section birth^32^, while another stated that preference of this type of birth was caused by concerns on physical changes during pregnancy and labor, fear of childbirth, positive attitudes towards obstetric technology, and the media as a source of information^34^.

In our study, it was determined that the stress and fear experienced by the participants in their first delivery room experience increased their traumatic childbirth perceptions significantly. While there are studies in the literature which reported that having taken a course on delivery affected the birth preferences of students positively (91.4%)^17^, it was also observed that, although students stated birth to be an experience that definitely needs to be felt, the processes of childbirth they witnessed during the delivery room practice, as part of their courses, increased their negative views. Provision of accurate information about childbirth for pregnant women and determination of their preferences of type of delivery are issues that are dependent on the knowledge levels of midwives. It is known that the views of midwifery students who are progressing on the path of becoming midwives on types of delivery before taking a course on delivery show similarities to those in the general public, while these views change after taking a course on delivery^35^. In this sense, it is clearly seen that, in order to positively influence the traumatic childbirth perceptions of students of midwifery and nursing, they need to be supported before their first delivery room experiences. Trainings in simulated environments before coming to the delivery room can contribute to this. It can reassure students to have someone more experienced with them in their first delivery room practice (their instructors, upper class students, mentor or another experienced midwife). The first day in the delivery room should be thus well planned.

In our study, the participants who had a history of difficult birth in their family were found to have higher levels of traumatic childbirth perceptions. A study on the childbirth perceptions of college students in Quebec determined that histories of family members (50.7% female, 39.9% male) affected the participants’ beliefs about childbirth^31^. Another study reported that the media and negative stories in the family are usually the factors that alter the attitudes of young women towards pregnancy and delivery^36^. Thomson et al.^37^ also found that fear of childbirth was affected by history of negative childbirth experiences among friends and family. As also mentioned in the literature, the findings support the idea that experiences of a difficult birth in the family show negative effects on women who have not experienced childbirth yet.

In our study, the indicators of traumatic birth perceptions were determined as satisfaction with the department of study, fear of childbirth, defining birth as a difficult and painful process and history of complicated birth in the family. Another study reported significant determinants of negative perceptions on childbirth as pregnancy planning, physical concerns, trait anxiety, expectation of labor pains, type of school and personal and professional sources of information^32^. Traumatic childbirth perceptions are higher among students who do not plan pregnancy, have higher levels of physical concerns and state trait anxiety, state anxiety and expectations of high labor pain. On the other hand, students who receive more information from personal and professional sources about childbirth and take courses in health sciences have lower levels of traumatic childbirth perceptions.

Among students, traumatic childbirth perceptions increase with high trait anxiety levels and low general self-esteem levels. Beliefs on self-efficacy also affect an individual’s style of thinking and emotional reactions. While individuals with high self-efficacy levels keep calm while facing difficult tasks and activities, those with low selfefficacy levels perceive events to be more difficult than they actually are and display a narrower point of view in the process of solving the problem^38^. Thus, low self-efficacy levels explain high traumatic childbirth perception levels.

## CONCLUSIONS

It was determined that traumatic childbirth perceptions increased as the levels of trait anxiety, increased and self-efficacy decreased. The traumatic childbirth perceptions were lower among those who were not afraid of childbirth, were satisfied with their department of study, considered childbirth to be a normal and happy event, preferred normal childbirth for the future, were comfortable in their first delivery room experience and had no history of complicated birth in their families. In this context, there is a need to define the negative points of view of midwifery and nursing students, assess their status of self-efficacy and anxiety that are affective on these perceptions and support such students. Professional information should be provided to the students with a positive approach, and guidance and support should be offered to them to help them develop autonomy in processes of inquiry, analysis, reflection of their knowledge into their clinical skills and decision-making, and reduce their traumas. Midwifery students should be given adaptation training in order to increase their clinical adaptation and supported by peer education from higher class midwifery students. Experience sharing trainings can be organized between experienced midwives and midwifery students in the clinic. Midwives who have positive childbirth perceptions will be more competent in reducing the negative feelings of the pregnant woman during the process of delivery.

## Data Availability

The data supporting this research is available from the authors on reasonable request.
